# The analysis of the internet of things database query and optimization using deep learning network model

**DOI:** 10.1371/journal.pone.0306291

**Published:** 2024-06-28

**Authors:** Xiaowen Ma

**Affiliations:** Library, Shandong University of Arts, Jinan, China; Amity University Haryana-Gurugram, INDIA

## Abstract

To explore the application effect of the deep learning (DL) network model in the Internet of Things (IoT) database query and optimization. This study first analyzes the architecture of IoT database queries, then explores the DL network model, and finally optimizes the DL network model through optimization strategies. The advantages of the optimized model in this study are verified through experiments. Experimental results show that the optimized model has higher efficiency than other models in the model training and parameter optimization stages. Especially when the data volume is 2000, the model training time and parameter optimization time of the optimized model are remarkably lower than that of the traditional model. In terms of resource consumption, the Central Processing Unit and Graphics Processing Unit usage and memory usage of all models have increased as the data volume rises. However, the optimized model exhibits better performance on energy consumption. In throughput analysis, the optimized model can maintain high transaction numbers and data volumes per second when handling large data requests, especially at 4000 data volumes, and its peak time processing capacity exceeds that of other models. Regarding latency, although the latency of all models increases with data volume, the optimized model performs better in database query response time and data processing latency. The results of this study not only reveal the optimized model’s superior performance in processing IoT database queries and their optimization but also provide a valuable reference for IoT data processing and DL model optimization. These findings help to promote the application of DL technology in the IoT field, especially in the need to deal with large-scale data and require efficient processing scenarios, and offer a vital reference for the research and practice in related fields.

## 1. Introduction

With today’s rapid development of information technology, the Internet of Things (IoT) technology has been widely used in industry, agriculture, medical care, transportation, and other fields, forming a massive dataset. These data are not only huge in volume but also have the characteristics of high dimension and real-time. Therefore, efficiently extracting valuable information from these massive datasets has become a major challenge in research and application [1–3]. In this context, database query and its optimization technology have become one of the key technologies in the data processing of IoT. As an advanced machine learning method, deep learning (DL) shows great potential in many fields, such as image recognition, natural language processing, and recommendation systems, because of its excellent data representation and feature learning ability. In recent years, researchers have begun to explore the application of DL technology in IoT database queries and optimization, aiming to improve query efficiency, reduce query latency, and optimize resource allocation to address various challenges in IoT data processing. However, DL models also face many challenges in handling IoT database queries and optimization, including but not limited to the complexity of model design and training, limited processing power for large-scale datasets, and insufficient model generalization ability. In addition, the dynamism and uncertainty of the IoT environment also pose new challenges to the deployment and maintenance of the model.

The research motivation is to study the application status and existing problems of existing DL network models in IoT database queries and analyze these models’ advantages and limitations in processing IoT data. The optimization method of the DL network model is explored to improve the model’s query efficiency, reduce resource consumption, and enhance the model’s adaptability and generalization ability to the dynamic data environment of IoT.

The principal contribution of this study lies in the proposition of an IoT database query optimization methodology predicated on DL models. Through the application of DL algorithms, the inherent relationships within the data are discerned and represented more effectively, thereby elevating the efficiency and accuracy of queries. Comparative assessments between the proposed DL-based approach and traditional query optimization methods are conducted across diverse query scenarios.

In summary, this study leverages the DL network model to optimize the querying process of IoT databases, with the overarching objective of refining both query efficiency and accuracy. The introduction of this method introduces novel perspectives and methodologies for the processing and utilization of IoT data, holding considerable theoretical and practical significance.

The structure of this study unfolds as follows: Section 1 elucidates the research background and motivation; Section 2 conducts a literature review and analyzes the limitations of prior studies; Section 3 introduces pertinent theories related to the DL model and IoT database; Section 4 performs comparative experiments on the optimized model. Section 5 synthesizes the experimental results and delineates the future trajectory of this study. This study can provide new solutions to the challenges of querying and optimizing IoT databases, making a substantive contribution to the effective management of IoT data.

## 2. Recent related work

Notable strides have been made within the realm of querying and optimizing IoT databases, as evidenced by several pertinent studies.

Andrade et al. (2023) proposed a machine learning-driven approach to optimize IoT data queries. Their methodology employed traditional machine learning algorithms to predict query performance, enhancing efficiency by reordering query plans. However, this technique fell short of fully harnessing the capabilities inherent in DL network models [4]. In a complementary endeavor, Vijayalakshmi and Karthika (2023) delved deeper into the optimization of IoT database queries, introducing a genetic algorithm-based approach. Employing genetic algorithms, they sought the optimal query plan and iteratively improved query performance. Yet, the scalability of this method may be compromised when handling extensive datasets [5]. Ahmad et al. (2023) proposed a novel deep reinforcement learning-centric optimization method for IoT database queries. Their approach involved designing a deep reinforcement learning model to iteratively learn the optimal query plan, thereby continually enhancing the query process. While this study maximized the potential of DL networks, challenges persisted in addressing queries involving heterogeneous data and real-time constraints [6]. Rinaldi et al. (2019) emphasized the escalating interest in IoT applications across diverse domains, investigating the impact of metadata variations on data extraction performance in Time Series & Spatial Temporal Databases (TSDBs). Their analyses revealed that the chosen data model significantly influenced database read-write performance, underscoring the critical role of data modeling [7]. Shankar and Maple (2023) introduced an attention-based methodology for optimizing IoT database queries, employing attention mechanisms to focus on crucial data features. While effective for handling complex IoT data, further enhancements were deemed necessary for managing large-scale datasets and high-concurrency queries [8]. Vinayakumar et al. (2020) concentrated on security concerns within IoT applications in smart cities, proposing a botnet detection system based on a two-level DL framework. This framework demonstrated substantial improvements in F1 performance, detection speed, and error rate [9]. Martino et al. (2019) asserted that DL has garnered considerable attention across diverse application fields. However, they noted a prevailing deficiency in the absence of a comprehensive processing structure capable of accommodating large-scale data processing, version control, and deployment without allegiance to any specific single-node framework. In response, the authors proposed an innovative higher-level framework. This framework employed database and model sequencing methodologies, facilitating the provision of unique and transformed data as required by the process. In order to exemplify the framework’s efficacy, the study employed a case study involving the high-speed control of power consumption within the large-scale IoT network of an optimized retail refrigeration system. Recognizing the inevitability of evolving data and requirements over time, the proposed framework demonstrated adaptability to future adjustments [10]. Liang (2019) contended that the extensive influx of data has posed challenges to the widespread implementation of DL in real-time processing within the IoT domain. In order to address this issue, the application of the singular value decomposition algorithm was recommended for DL preprocessing of large-scale data input. Additionally, the finite memory subspace optimization algorithm was employed for substantial data input, resulting in a notable enhancement of data processing speed. The outcomes indicated that both the singular value decomposition algorithm and the finite memory subspace optimization algorithm could significantly reduce DL neural network inputs without compromising performance, thereby substantially improving the data processing speed of DL-based IoT applications [11]. In a study by Liu et al. (2020), a user-centered ultra-dense network was proposed. Subsequently, a security system tailored to this architecture was designed, leveraging the Back Propagation Neural Network (BPNN). Addressing the challenge of secure data transmission between network entities, a lightweight data security transmission algorithm based on the implicit certificate was introduced. This algorithm utilized a lightweight implicit certificate to generate a temporary session key through a reconfigurable public-private key pair, facilitating the encryption and protection of transmitted data. The solution effectively resolved data security transmission concerns at the access node [12]. Cura et al. (2021) contributed by developing a neural network model founded on Long Short-Term Memory (LSTM) and Convolutional Neural Network (CNN) architectures for the classification and assessment of bus driver behaviors. The behaviors under scrutiny included deceleration, engine speed pedal manipulation, turning maneuvers, and lane-changing attempts. Test scenarios encompassed concrete paved tracks for deceleration, engine speed, and turning, while lane-change assessments were conducted on a commercial asphalt highway. The CNN architecture exhibited superior performance in identifying aggressive driving compared to the LSTM network equipped with behavior modeling [13].

Earlier investigations indicate that certain studies inadequately exploit the benefits offered by DL network models, often falling short in effectively utilizing correlated information within extensive and heterogeneous IoT datasets. Moreover, persistent limitations exist in addressing the challenges posed by large-scale data and high-concurrency queries. This underscores the need for continual improvements in system scalability and concurrency performance. The pervasive adoption of DL models in IoT databases has led to the formulation of various optimized reference methods. This present inquiry seeks to extend the application of DL to image facial emotion recognition, building upon the achievements of previous studies, with the aim of providing additional data-driven support to this specific domain. The innovation of this study is mainly reflected in the following aspects. Firstly, the DL technology is applied to optimize IoT database queries, identifying and analyzing features and patterns in IoT data through DL models, effectively improving query efficiency and accuracy. Secondly, in response to the characteristics of IoT data, this study introduces an innovative DL algorithm specifically designed and optimized, which can more accurately process and analyze large-scale, heterogeneous, and dynamically changing IoT data. In addition, this study not only proposes new methods in theory, but also tests and validates them in practical IoT environments, proving their practical application value and reflecting the close integration of theory and practice. Through experimental results, this study demonstrates significant improvements in query efficiency, resource consumption, throughput, and latency of the optimized DL model, especially in handling large-scale data. Finally, this study not only reveals the excellent performance of optimization models in handling IoT database queries, but also provides valuable references for IoT data processing and DL model optimization, promoting the application and development of DL technology in the field of IoT. Through these innovative points, this study has significant breakthroughs in theory and methods, and also demonstrates its excellent performance in IoT database query optimization through practical verification, providing new ideas and solutions for research and practice in related fields.

## 3. Research theory and method

### 3.1. IoT database

**Fig 1:**
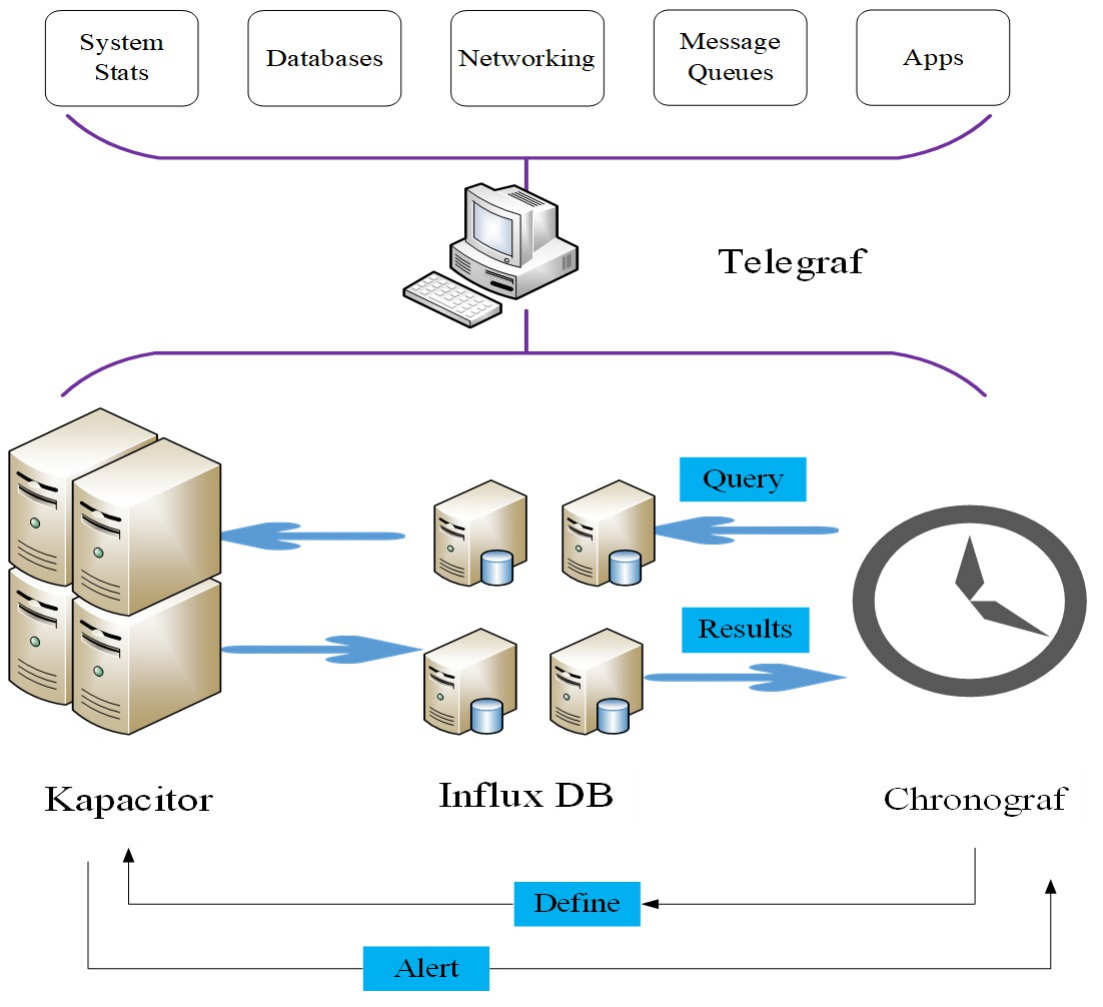
Composition structure diagram of IoT database.

The IoT database comprises diverse components encompassing sensors and actuators, connectivity infrastructure, visualization mechanisms, computing resources, security protocols, analytical tools, and cloud-based platforms [14–17], as depicted in Fig 1.

The foundational components of the IoT encompass sensors and actuators, connectivity infrastructure, computing mechanisms, cloud-based platforms, analytical tools, visualization technologies, and security protocols.

Sensors play a pivotal role in sensing and collecting diverse data pertaining to the physical environment, including variables such as temperature, humidity, and light intensity [18]. The actuators execute requisite actions or operations predicated on external instructions or perceived data. Sensors and actuators constitute pivotal components within the IoT, facilitating the acquisition of environmental information and subsequent responsive actions within IoT systems. The connectivity of devices in the IoT necessitates integration through diverse networks, culminating in the establishment of an extensive network system [19, 20]. These connections, whether realized through wireless or wired technology, empower IoT devices to engage in communication and data exchange, thereby fostering the development of an intelligent and interconnected system.

IoT devices commonly necessitate a certain degree of local computing capacity to process extensive data volumes or execute intricate algorithms. This computational task may be performed directly on the IoT device or via edge computing methodologies, mitigating reliance on network transmission and enhancing data processing efficiency [21]. Regarding cloud-based platforms, IoT predominantly leverages these platforms for the storage, management, and analysis of substantial device-generated data. Such platforms exhibit high scalability and robust computing and storage capabilities, enabling users to upload device data to the cloud. This facilitates the extraction of valuable insights through analysis and mining, subsequently driving informed business decisions and fostering innovation [22–25]. The substantial influx of data from the IoT necessitates proficient analysis and exploration to uncover latent patterns, trends, and correlations. Through data analysis, profound insights into device functionality, user behavior, market dynamics, and more can be extracted, providing a scientific foundation for decision-makers. Visualization technology is widely embraced to enhance the understanding and utilization of IoT data. Transforming abstract data into intuitive charts, graphs, and dashboards enables users to comprehend and analyze information swiftly, facilitating prompt decision-making. Security assumes a critical role in the IoT landscape, given its involvement with numerous devices, networks, and data streams. IoT security protocols encompass device authentication, encrypted communication, authentication mechanisms, and data privacy protection. Safeguarding the security of IoT systems is imperative to prevent unauthorized access or misuse of devices and data [26].

In summation, the foundational and functional elements of IoT encompass sensors and actuators, connectivity, computing capabilities, cloud-based platforms, analytics, visualization tools, and security measures. These components collaborate synergistically within the IoT system, propelling ongoing development and innovation in IoT technology.

### 3.2. The DL model

As a classical Artificial Neural Network (ANN) algorithm, the BPNN executes error reverse transmission through a backpropagation algorithm during the training process. It continually adjusts weights and biases to minimize the loss function, demonstrating proficiency in handling nonlinear problems and tasks such as pattern recognition [27]. Nonetheless, limitations arise in managing sequence data and addressing long-term dependencies, necessitating the consideration of more advanced algorithms.

DL constitutes a neural network-based machine learning paradigm that assimilates and represents intricate patterns and underlying structures through multi-layered, stacked networks of neurons. Leveraging deep feature representation and automatic learning capabilities, DL has exhibited notable success in computer vision, natural language processing (NLP), and various domains. Utilizing technologies like CNN and Recurrent Neural Network (RNN), models can be trained on extensive datasets to extract effective feature representations, achieving high-performance tasks such as classification, regression, and generation. CNN, specifically designed to handle grid-type data, employs the convolutional layer, pooling layer, and fully connected layer to extract spatial features in images effectively. It excels in image recognition, classification, and segmentation by leveraging features such as local spatial information and weight sharing. Thus, CNN has become a preferred algorithm in image-processing tasks, making significant breakthroughs in computer vision [28–30].

RNN stands out as a neural network model proficient in handling sequential data, owing to its dynamic structure that facilitates the transmission of information from the preceding time step to the subsequent one. RNN finds widespread applications in NLP, speech recognition, time series analysis, and various other domains. Nevertheless, traditional RNNs encounter challenges such as gradient vanishing and explosion, posing difficulties in capturing long-term dependencies inherent in sequential data. To address this predicament, LSTM is introduced. This specialized form of RNN excels in managing long-term dependencies, enhancing the performance of RNN in modeling sequential data.

LSTM serves as a distinctive RNN variant that effectively mitigates issues like gradient vanishing and explosion prevalent in traditional RNN through the incorporation of memory units and gating mechanisms. This innovation empowers the LSTM model to adeptly capture long-term dependencies within sequential data by dynamically discarding and updating information. Noteworthy achievements of LSTM include significant contributions to language modeling, machine translation, and music generation, establishing its widespread adoption in tasks demanding processing time series or sequential data.

### 3.3. Construction of the optimized model

The key to the model optimization strategy is to improve the accuracy, efficiency, and generalization ability of the model to better deal with the characteristics of IoT databases, such as large-scale data, high dimensionality, and real-time. Specific optimization strategies are exhibited in Table 1.

**Table 1:** Optimization strategy.

Dimension	Optimization strategy
Data preprocessing and enhancement	Data cleaning, feature engineering, data augmentation
Model architecture adjustment	Lightweight models, multitasking learning, model pruning, and quantization
Training techniques	Transfer learning, data imbalance handling, regularization, and early stopping
Efficient training and query execution	Distributed training, caching strategy, efficient index structure
Model evaluation and tuning	Cross-validation, hyperparameter optimization, performance monitoring

**Fig 2:**
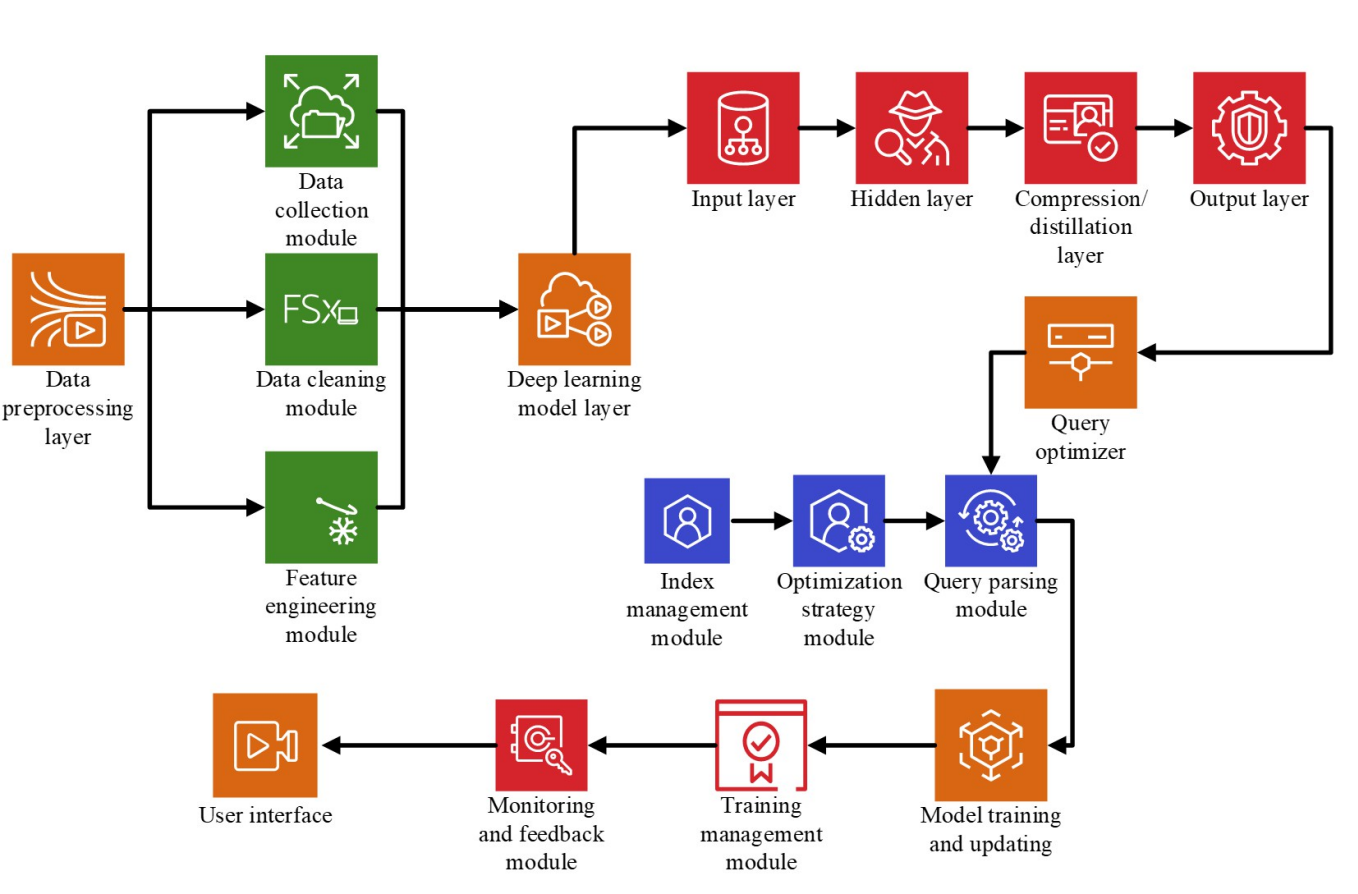
Optimized model architecture.

Through these optimization strategies, the DL model’s performance in IoT database queries and optimization tasks can be effectively improved while ensuring the model’s scalability and practicality. The optimized model architecture is presented in Fig 2.

This architecture combines data preprocessing, DL modeling, query optimization, and user interface closely, which improves the efficiency and accuracy of IoT database queries and ensures the maintainability and scalability of the system. According to specific application scenarios and requirements, this architecture can be adjusted and optimized accordingly. The code for running the model is as follows:

def load_and_preprocess_data(file_path):

      data = pd.read_csv(file_path)

      features = data.drop(’target’, axis = 1).values

      target = data[’target’].values

      scaler = StandardScaler()

      features_scaled = scaler.fit_transform(features)

      X_train, X_test, y_train, y_test = train_test_split(features_scaled, target, test_size = 0.2, random_state = 42)

      X_train = X_train.reshape((X_train.shape[0], X_train.shape[1], 1))

      X_test = X_test.reshape((X_test.shape[0], X_test.shape[1], 1))

      return X_train, X_test, y_train, y_test

### 3.4. Experimental data source and environment

The simulation training is executed using MATLAB 2018a on a system running Windows 8, equipped with an Intel Core i5 processor, 4GB of memory, and a 64-bit operating system.

The dataset selected for the experiment is the IoT dataset in the UCI Machine Learning Repository, a library of widely collected and publicly available machine learning datasets. These datasets cover multiple application scenarios in fields ranging from finance and healthcare to IoT, making them an ideal resource for scientific research, education, and laboratory experiments. At the same time, a variety of IoT-related datasets are provided, involving environmental monitoring, smart home, health monitoring, and other fields, and individuals can choose the most suitable data according to the specific research direction. The dataset can be downloaded through the website (https://archive.ics.uci.edu/). To ensure the effectiveness of the experiment, the parameters of the model are set uniformly. The network architecture comprises a single hidden layer with 32 neurons. A uniform learning rate of 0.001 is employed for both models, with a training duration of 128 iterations. Furthermore, the Sigmoid activation function is uniformly applied to both models.

## 4. Simulation results and analysis

### 4.1. Model performance comparison experiment

**Fig 3:**
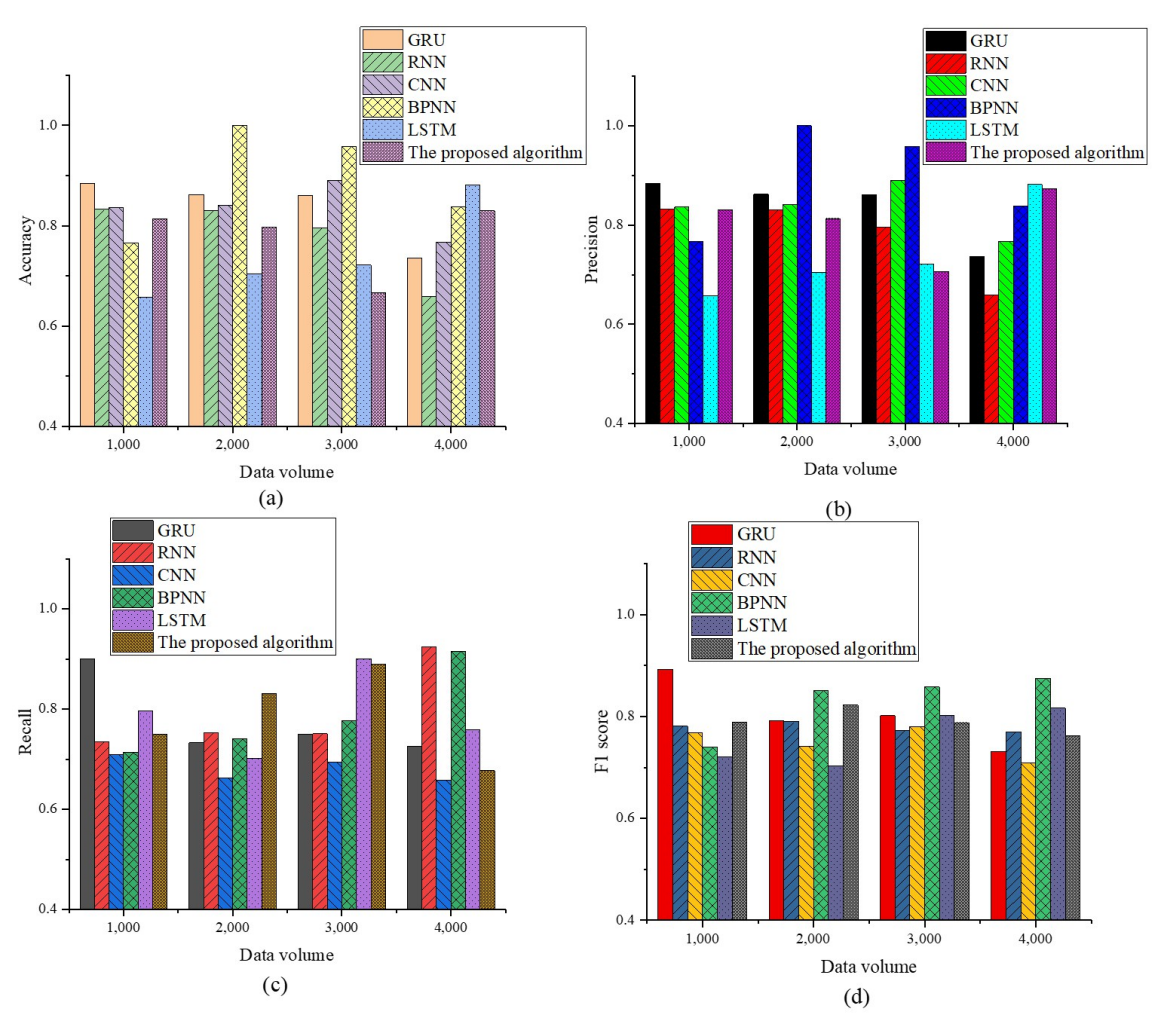
Comparison of training accuracy of different algorithms (a)Accuracy; (b)Precision; (c) Recall; (d) F1 score.

The models compared in the experiment include Gated Recurrent Unit (GRU), RNN, CNN, BPNN, and LSTM. The comparison indicators encompass accuracy, precision, recall, and F1 score. The performance comparison results are depicted in Fig 3.

According to the results in Fig 3A, 3B, 3C and 3D, it can be observed that in terms of accuracy, the optimized model proposed in this study exhibits certain volatility under different data volume settings. In particular, at 1000 and 4000 data volume settings, the model presents high accuracy of 0.8143 and 0.8298, respectively, indicating that the optimized model can maintain high performance when dealing with both smaller and larger datasets. However, at a data volume setting of 3000, the accuracy of the optimized model drops to 0.6673, which may imply that the model’s performance may suffer at some specific data sizes or characteristics. In most data volume settings, the optimized model demonstrates competitiveness and advantages over other models. Especially compared with the LSTM model, the optimized model has higher accuracy than the LSTM model at the 4000 data volume setting. In terms of precision, the optimized model attains better precision than other models in most cases, especially when the data volume is set at 4000, the optimized model reaches 0.8733, illustrating that this model has good performance when dealing with large-scale data. Considering recall, the optimized model reveals a good recall in most cases, especially under the data volume settings of 2000 and 3000, the recall reaches 0.8317 and 0.8906, respectively, showing that under these data scales, the optimized model has strong performance. However, it is worth noting that the performance of all models fluctuates with various data volumes, reflecting the impact of different data volumes on model performance and the ability of models to adapt to diverse data scales. The optimized model displays a relatively high F1 score under most data volume settings, especially the F1 score of 0.8223 for 2000 data volume, showing that when dealing with medium-scale data, the optimized model has excellent performance and a good balance of accuracy and recall. In some cases, such as at the 4000 data volume setting, other models (especially BPNN and LSTM) have higher F1 scores. Overall, the optimized model demonstrates its potential to maintain outstanding performance in various situations.

### 4.2. Model efficiency comparison experiment

**Fig 4:**
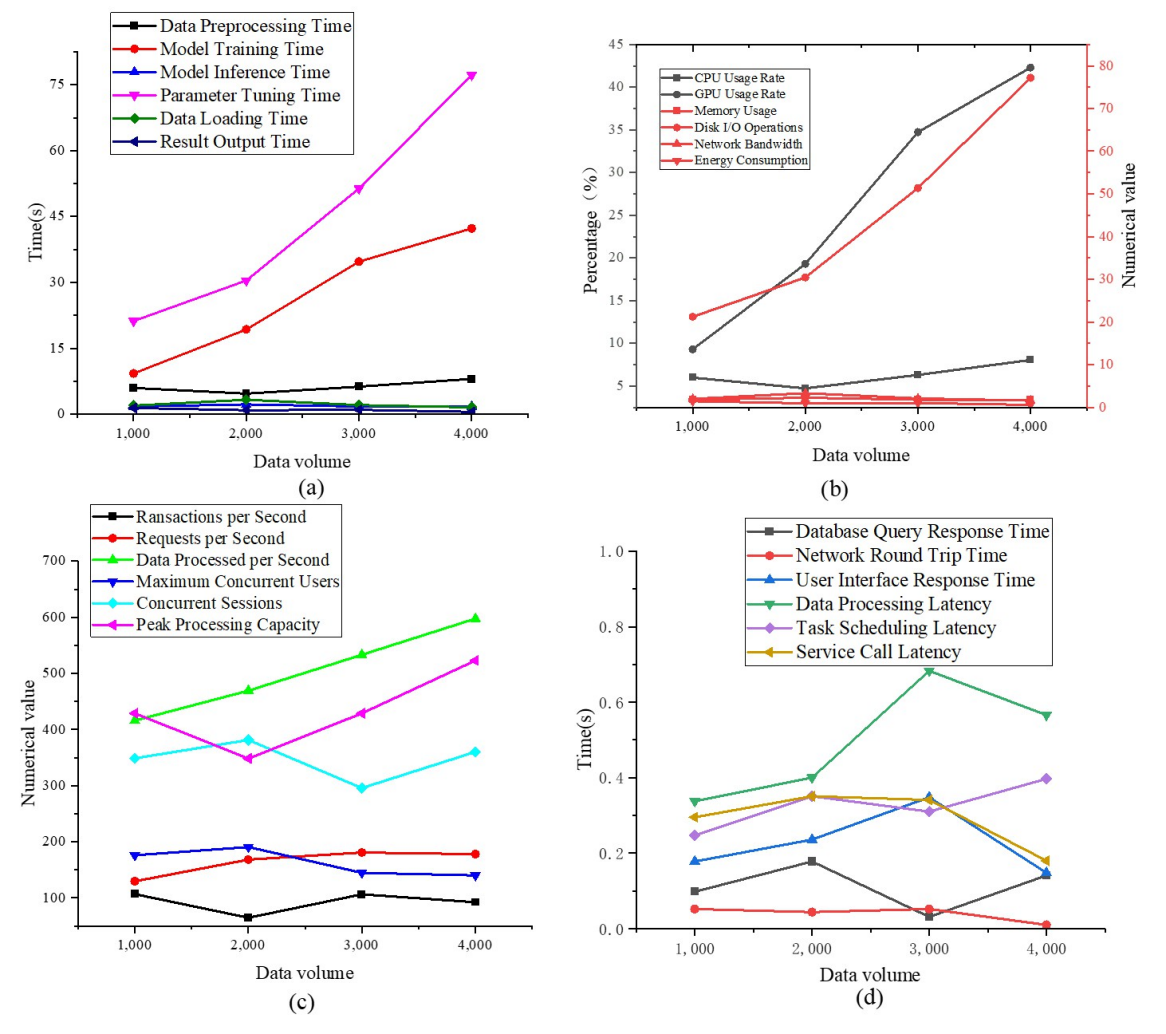
Comparison results of model efficiency (a) Computation Time; (b) Resource Consumption; (c) Throughput; (d) Latency.

To further validate the advantages of the optimized model, four indicators are selected for comparison: computation time, resource consumption, throughput, and latency. The experimental results are denoted in Fig 4.

Fig 4A, 4B, 4C and 4D reveal that in terms of calculation time, at a data volume of 1000, the data preprocessing time is 5.99 seconds; When the data volume increases to 4000, the preprocessing time increases to 8.05 seconds. This means that the data preprocessing time slightly rises with the increase in data volume, but the growth rate is not fast, indicating that the preprocessing step is relatively stable in terms of time consumption compared to the entire processing process. For model training time, it can be found that it increases significantly from 9.30 seconds for a data volume of 1000 to 42.30 seconds for a data volume of 4000. Model training time increases remarkably with the rise in data volume, which is in line with expectations, as larger datasets typically require more time to complete the model training process. Parameter tuning time is 21.21 seconds for a data volume of 1000, notably increasing to 77.19 seconds for a data volume of 4000. This marked growth indicates that the parameter tuning process is highly sensitive to data volume, especially when dealing with large data volumes, the time required to find the optimal parameter configuration significantly increases. The result output time slightly decreases from 1.44 seconds to 0.57 seconds for a data volume of 1000 to 4000. Considering resource consumption, the CPU usage rate is approximately 44.56% for a data volume of 1000, increasing to 53.76% for a data volume of 4000. The CPU usage rate slightly increases as data volume is added, illustrating that as the amount of processed data increases, the CPU’s computational tasks also increase accordingly. The GPU usage rate is about 30.99% for a data volume of 1000, prominently increasing to 67.78% for a data volume of 4000. This remarkable increase may indicate that for tasks requiring a large amount of parallel computation, the utilization of the GPU distinctly increases with the rise in data volume. The memory usage fluctuates at diverse data volumes, decreasing from approximately 3.98GB to 2.17GB for a data volume of 1000 to 4000. This fluctuation may be due to different memory requirements at various stages of the data processing process or as a result of randomness in the simulated data. In practice, memory usage is usually positively correlated with the data volume being processed. The frequency of disk I/O operations exhibits a nonlinear pattern, decreasing from 106 times to a minimum of 50 times for a data volume of 1000 to 2000, then increasing again for data volumes of 3000 and 4000. This variability in disk I/O operations reflects the changing demands for data read/write operations when processing data of varying scales. Concurrently, the speed of disk I/O operations shows a declining trend, from approximately 23.69MB/s to 18.49MB/s for a data volume of 1000 to 4000. This decrease in disk I/O consumption may be attributed to task-specific factors, such as increased local computations, reducing the necessity for data transmission. Energy consumption demonstrates an upward trend, rising from approximately 126 watt-hours for a data volume of 1000 to approximately 252.86 watt-hours for a data volume of 4000. This escalation in energy usage corresponds to the augmented demand for computational resources and time required to process larger datasets, consequently consuming more energy. Regarding throughput, the optimized model processes approximately 106.87 transactions per second for a data volume of 1000, with a slight reduction to 92.30 for a data volume of 4000. This indicates a marginal decline in the system’s transaction processing capability with larger data volumes, albeit maintaining overall efficiency. Concurrently, the number of requests processed per second rises from around 129.69 to 177.94 for a data volume of 1000 to 4000, suggesting enhancements in the system’s capacity to handle concurrent requests. As data volume increases, the data volume processed per second also rises, from 416.08MB to 597.55MB. This improvement in this metric indicates that the system can effectively process and analyze increasingly large data volumes, demonstrating good data processing capabilities. The maximum number of concurrent users and the number of sessions that the system can maintain at the same time, these two metrics that show a certain fluctuation as the data volume increases. The system demonstrates the ability to support numerous users and concurrent sessions online, ensuring a satisfactory user experience and system responsiveness. Peak processing capacity displays fluctuations with increasing data volume, initially decreasing slightly from 428.93 to 348.39, then increasing to 523.04. These variations may stem from dynamic resource allocation and optimization within the system to accommodate diverse workload demands. Latency exhibits fluctuations in database query response times as data volume increases, ranging from 0.098 seconds to 0.178 seconds, with an outlier at a data volume of 3000 (adjusted to 0 seconds), followed by a subsequent increase to 0.141 seconds. These fluctuations may indicate alterations in query optimization or index efficiency at different data scales. Overall, the network round-trip time (RTT) remains at a low level, decreasing from 0.052 seconds to 0.044 seconds, then increasing to 0.052 seconds, but showing an apparent decrease at a data volume of 4000 (to 0.010 seconds). This may indicate that network conditions or server responses can be optimized in some cases, reducing communication latency. User interface response time and data processing latency: These two metrics increase with the growth in data volume, rising from 0.178 seconds and 0.338 seconds to 0.348 seconds and 0.683 seconds respectively. This indicates that the efficiency of both the user interface and data processing may be impacted when handling larger volumes of data.

## 5. Conclusion

In this study, the performance of the optimized DL network model in IoT database queries and its optimization aspects are explored through experiments, particularly focusing on four key dimensions: computation time, resource consumption, throughput, and latency. By comparing the performance of GRU, RNN, CNN, BPNN, LSTM, and the proposed optimized model under different data volumes, the optimized model demonstrated higher efficiency in model training time and parameter tuning time compared to traditional models, especially when the data volume is 2000. The optimized model’s parameter tuning time and model training time are markedly lower than other models, showing the effectiveness of optimization measures in accelerating the model training and tuning process. In terms of resource consumption, it can be observed that with the increase in data volume, the CPU usage, GPU usage, and memory usage of all models are increased. However, the optimized model exhibits better performance in energy consumption, especially when the data volume is 4000, showing higher energy efficiency than other models. Throughput data illustrate that the optimized model can maintain a higher number of transactions per second and data volume when processing large-scale data, especially when the data volume is 4000, its peak processing capacity exceeds other models, demonstrating its strong ability to handle large-scale data requests. The analysis of latency indicates that all models experience an increase in latency as data volume increases, the optimized model presents lower latency in database query response time and data processing delay. Especially when the data volume is set to 3000 and 4000, the optimized model’s user interface response time and service invocation latency are lower than other models. These findings not only validate the effectiveness of the optimization measures proposed here but also offer valuable references and insights for future research on DL network models in IoT database queries and optimization. Future work will further explore other aspects of model optimization, such as reducing energy consumption and improving the system’s real-time response capability, to meet the growing demand for IoT data processing.

## Supporting information

S1 Data (XLSX)
